# (Patho)Physiology of Glycosylphosphatidylinositol-Anchored Proteins I: Localization at Plasma Membranes and Extracellular Compartments

**DOI:** 10.3390/biom13050855

**Published:** 2023-05-18

**Authors:** Günter A. Müller, Timo D. Müller

**Affiliations:** 1 Institute for Diabetes and Obesity (IDO), Helmholtz Diabetes Center (HDC) at Helmholtz Zentrum München, German Research Center for Environmental Health (GmbH), Ingolstädter Landstraße 1, 85764 Oberschleissheim, Germany; timo.mueller@helmholtz-munich.de; 2 German Center for Diabetes Research (DZD), 85764 Oberschleissheim, Germany

**Keywords:** adipose cells, extracellular vesicles, glycosylphosphatidylinositol (GPI)-anchored proteins (GPI-APs), (G)PI-specific phospholipase D (GPLD1), metabolic diseases, protein release, sulfonylurea drugs

## Abstract

Glycosylphosphatidylinositol (GPI)-anchored proteins (APs) are anchored at the outer leaflet of plasma membranes (PMs) of all eukaryotic organisms studied so far by covalent linkage to a highly conserved glycolipid rather than a transmembrane domain. Since their first description, experimental data have been accumulating for the capability of GPI-APs to be released from PMs into the surrounding milieu. It became evident that this release results in distinct arrangements of GPI-APs which are compatible with the aqueous milieu upon loss of their GPI anchor by (proteolytic or lipolytic) cleavage or in the course of shielding of the full-length GPI anchor by incorporation into extracellular vesicles, lipoprotein-like particles and (lyso)phospholipid- and cholesterol-harboring micelle-like complexes or by association with GPI-binding proteins or/and other full-length GPI-APs. In mammalian organisms, the (patho)physiological roles of the released GPI-APs in the extracellular environment, such as blood and tissue cells, depend on the molecular mechanisms of their release as well as the cell types and tissues involved, and are controlled by their removal from circulation. This is accomplished by endocytic uptake by liver cells and/or degradation by GPI-specific phospholipase D in order to bypass potential unwanted effects of the released GPI-APs or their transfer from the releasing donor to acceptor cells (which will be reviewed in a forthcoming manuscript).

## 1. Introduction

**Figure 1:**
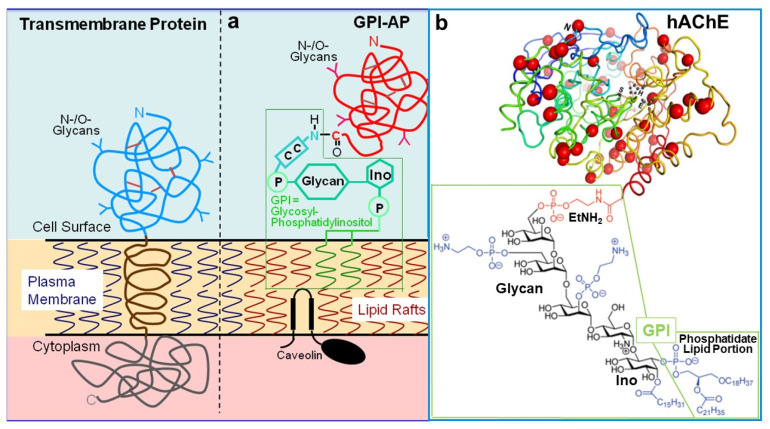
(**a**) Schematic structures of typical mammalian GPI-APs and of canonical and atypical transmembrane proteins. (right section) GPI-APs consist of a large protein domain (red), often with N-/O-glycosidically linked carbohydrates (blue) and intrachain disulfide bonds (brown), and a GPI anchor which is constituted of phosphatidylinositol (P-Ino) with long saturated fatty acids (dark green) and a glycan core (light green). The carboxy-terminus of the protein domain and the terminal (third) mannose residue of the glycan core are coupled through ethanolamine via amide (H-N-C=O) and phosphodiester (-P-) bonds, respectively. The fatty acids of the GPI anchor are embedded in the outer leaflet of the PM bilayer, often at lipid rafts, which typically harbor cholesterol and caveolin-1. Caveolin-1 represents an atypical monotopic transmembrane protein with both termini facing the cytoplasm. (left section) The transmembrane protein shown is a typical class I representative, with the amino- and carboxy-terminal polypeptide domains protruding into the extracellular space and cytoplasm, respectively, separated by the α-helical transmembrane domain. (**b**) Molecular structure of the typical GPI-AP human acetylcholinesterase (hAChE). (upper section) Crystal structure of wildtype and mutant AChE generated using the “Protein Repair One-Stop Shop” program. The cartoon representation of the crystal structure of hAChE displays the mutated residues in the designed variant. hAChE is shown as a backbone trace, color-coded from blue at the amino-terminus (labeled N) to red at the carboxy-terminus (labeled C). The residues of the catalytic triad, Ser-203, His-447 and Glu-334 are displayed as black ball and stick models, and labeled S, H and E, respectively. Fifty-one mutated residues distributed throughout the sequence as generated by Goldenzweig and coworkers [15] are highlighted as red spheres (adapted from ref. [16]). (lower section) Structure of its GPI anchor according to Deeg and coworkers [17]. The three constituents of the GPI anchor (enclosed by green lines) are the EtNH_2_-P bridge (red), the highly conserved glycan core (black) and the phosphatidate lipid portion (blue), as it is cleaved off by GPI-specific phospholipase D (see below). The presence of additional acyl or alkyl chains at the lipid portion as well as EtNH_3_^+^-P moieties at the glycan core (in blue) is variable. Removal of the EtNH_3_^+^-P side branches by PGA5P phosphodiesterase is required for efficient recruitment into ER-exit sites (ERES) and subsequent trafficking from the endoplasmic reticulum to the Golgi apparatus (see below, Section 2).

In eukaryotic cells, from yeast to mammalian cells, a specific class of surface proteins is anchored at the outer phospholipid layer of plasma membranes (PMs) via a glycosylphosphatidylinositol (GPI) glycolipid moiety. In mammalian organisms, GPI-APs encompass about 3–5% of the total PM proteins, i.e., about 150 representatives [1,2,3]. The protozoal GPI-APs variant surface glycoprotein (VSG), merozoite surface antigen and promastigote surface protease from the parasites *Trypanosoma brucei* [4], *Plasmodium falciparum* [5] and *Leishmania major* [6], respectively, were the first to have their structure of their anchor elucidated. They are made of a highly conserved hydrophilic glycan core consisting of a non-acetylated glucosamine (GlcN) and three mannose (Man) residues connected via specific glycosidic linkages, which at one end is glycosidically coupled to amphiphilic phosphatidylinositol (PI) (typically diacyl-PI, exclusively, or a mixture of diacyl and 1-alkyl-2-acyl-PI) and at the other non-reducing end, the third mannose, or in rare cases the second mannose, is invariably amide-linked to the carboxy-terminus of the protein moiety via a phosphoethanolamine (EtN-P) bridge [7,8]. This GPI glycan core structure is highly conserved from yeast to protozoa to humans (Figure 1). It can be modified in both mammalian cells, protozoa and fungi by addition of various carbohydrate or acyl moieties (for a review, see [9,10,11]) or by remodeling of the fatty acids (for a review, see [12]), which has meanwhile become amenable to detection by sophisticated analytical methods [13,14].

In particular, in yeast the GPI glycan precursor has the structure Man-(EtN-P)Man-(EtN-P)Man-(EtN-P)Man-GlcN, which after its fabrication at the endoplasmic reticulum (ER) undergoes remodeling in the course of trafficking of the GPI-AP to the PMs. This involves the coupling of a fifth Man residue to the fourth Man residue by certain mannosyltransferases, which have escaped identification so far, and the elimination of the side chain EtN-P of the first and second Man residues by the phosphodiesterases Cdc1 and Ted1, respectively [11,13,18,19]. Furthermore, during infections by protozoa, such as in toxoplasmosis, malaria, and trypanosomiasis, GPI glycolipids actively modulate the host immune system [20]. The PMs of the parasites causing those diseases are known to harbor multiple copies of a particular GPI-AP (and free GPI lipid) which cooperate in the formation of a coat for the protection from infection or as immunomodulators. In vitro and in vivo studies have demonstrated that various modifications of protozoal and fungal GPI glycans elicit a variety of distinct and specific immune responses. For instance, galectin-3-dependent interaction of parasitic GPI glycans with macrophages leads to stimulation of TNF-α synthesis [21]. Similarly, immunization with galactose-harboring GPI glycan of VSG triggers TNF-α production in mice, resulting in their extended life span upon infection with trypanosomes [22]. Elucidation of the structure of the GPI glycan of *Toxoplasma gondii* and *Trypanosoma congolense* has revealed the glycosidic linkage of *N*-acetyl-galactosaminyl as well as glucosyl-*N*-acetyl-galactosaminyl and galactosyl-*N*-acetyl-glucosaminyl side branches, respectively, to the first mannose residue of the glycan core [23]. In trypanosomes, an unusual ß1-6 *N*-acetyl-glucosaminyl-transferase has been suggested to catalyze the modification of the glycan core of their GPI anchors [24].

## 2. Biogenesis and Expression at PMs of GPI-APs

GPI-APs are produced by coupling of the completed GPI anchor, prefabricated by stepwise transfer from activated precursors of the corresponding carbohydrate and EtN-P residues to PI at the luminal face of the ER membranes, to the carboxy-terminus of the polypeptide precursor moiety upon its translation and transient arrest at the ER membranes (for a review, see [25,26]). Total synthesis of the GPI anchor in mammalian cells requires 13 reactions catalyzed by more than 23 gene products, among them for transfer and amide coupling of the GPI anchor to the polypeptide moiety the membrane-bound GPI transamidase (GPI-T), including the catalytic subunits PIG-K and GPAA1 and the regulatory subunits PIG-S, PIG-T and PIG-U in mammals [27], including humans [28], and their homologues in Drosophila [29] and yeast (for instance GPI8 as PIG-K homolog) (for a review, see [30,31,32,33]). Consequently, the polypeptide precursor is equipped with two signals, a GPI attachment signal sequence at their carboxy-terminus recognized by the GPI-T [34,35,36] and a typical secretory pathway signal sequence at their amino-terminus for translocation into and quality control (and degradation) at the ER membranes [37,38,39,40] and subsequent transport to the PMs [41]. Both signals consist of stretches of hydrophobic amino acids [35] and are subsequently clipped off by signal peptidase and GPI-T, respectively. Unexpectedly, the canonical signal recognition particle-dependent pathway engaged by the majority of membrane and secretory proteins is not involved in the translocation of GPI-APs across the ER membranes, which instead depends on a network of cytosolic proteins and factors [42]. For the prediction of GPI anchorage of cell surface proteins, algorithms such as Big-PI [1], GPI SOM [36] and FragAnchor [3], as well as web-based prediction tools [43,44,45,46], are available. Importantly, the entire information required and adequate for “glypiation”, i.e., the post-translational addition of GPI anchors, seems to be contained in the GPI attachment signal sequence and is decoded by GPI-T, since it manages to transform soluble proteins into the corresponding GPI-AP upon recombinant addition to the carboxy-terminus, as has been exemplified with numerous secretory protein-GPI fusion constructs (e.g., ref. [47]). Interestingly, subunits of the GPI-T, such as PIG-U in mammals, as well as certain GPI-APs, have been recognized to be involved in the initial stages of tumor development and tumor progression and may be used as putative biomarkers for specific diagnosis, therapy and prognosis [48].

The data available demonstrate that soon after coupling of the GPI anchor to the protein moiety and in the course of transport of the assembled GPI-AP along the secretory pathway to the cell surface, the lipid portion of the GPI anchor is subjected to structural remodeling which is critical for proper functioning and intracellular trafficking (for a review see [12,25,31]). For instance, in the yeast *Saccharomyces cerevisiae*, lipid remodeling has been shown to happen at the ER, starting with deacylation of the GPI inositol moiety by Bst1 (orthologue to mammalian PGAP1) [49], a process thought to confer quality control for correct GPI assembly, continued by removal of an unsaturated fatty acid at the sn-2 position by Per1 phospholipase A_2_ (orthologue to mammalian PGAP3) and subsequent coupling of a very-long-chain saturated fatty acid (C26) by Gup1 reacylase (not orthologue to mammalian PGAP2, but belonging to the mammalian MBOAT family of acyltransferases) [50] and then in the majority of yeast GPI-APs finished by replacement of the C26 diacylglycerol with ceramide, which also harbors a very-long-chain saturated fatty acid by Cwh43 (the amino- and carboxy-terminal parts corresponding to mammalian PGAP2 and PGAP2-interacting protein p24, respectively; for a review, see [25]) [51,52].

Interestingly, yeast and mammalian cells differ in the sites where GPI-APs become sorted from typical secretory proteins, with ER operating as the major site of sorting for the former and the Golgi apparatus for the latter (for a review see [25]). Importantly, in yeast, typical ceramide-harboring GPI-APs undergo clustering into discrete ER zones which are equipped with specific ER exit sites (ERES) [53]. Very recent studies indicate that in yeast ceramide remodeling is a prerequisite for clustering at the ER of GPI-APs and their resulting subsequent sorting, trafficking to PMs and insertion into lipid rafts. In greater detail, it has become apparent that a GPI-AP upon its remodeling by Cwh43 becomes recognized by the GPI-glycan remodelase Ted1 in order to facilitate its interaction with the p24 complex and in consequence its export from the ER, as part of a quality control mechanism of GPI-AP cargo sorting at the level of the ER [53]. It is tempting to speculate that GPI anchors harboring C26 ceramide associate with free C26 ceramide present in the ER membranes to produce ceramide-enriched lipid rafts, which possibly constitute interdigitated phases resulting in segregation of the GPI-APs from transmembrane proteins [54].

In addition to remodeling of the lipid portion of the GPI anchor, its glycan portion is also subjected to another modification process prior to exit from the ER. It has been demonstrated that after elimination of the EtN-P side chain, which had initially been attached to the second mannose residue of the glycan core, by a specific phosphodiesterase, encoded by Ted1 and Cdc1 in yeast [55] and PGA5P/MPPE1 in mammalian cells [56], the yeast transmembrane cargo receptor p24 associates with the remodeled glycan for subsequent specific recruitment of Lst1, which represents a specific isoform of the major COPII cargo interacting subunit Sec24 [56] and mediates the biogenesis of COPII vesicles enriched in GPI-APs for their sorting at ERES in *Saccharomyces cerevisiae* [55,57]. On the basis of the finding that in PGAP5-deficient mammalian cells GPI-APs did not efficiently enter into ERES, it has been suggested that PGAP5- and Ted1-mediated removal of the EtN-P side chain is a prerequisite for productive recruitment of GPI-APs into ERES and subsequent trafficking from the ER to the Golgi apparatus in both mammals [58] and yeast [56,57], respectively.

In mammalian cells, upon arrival of GPI-APs at the Golgi apparatus, the unsaturated fatty acyl chains in 1-alkyl-2-acylglycerols at the sn2 position as well as in diacylglycerols of the GPI anchor are exchanged for stearic acid by the action of the phospholipase A_2_ PGAP3, an orthologue to yeast Per1 [59]. During this remodeling, lyso-intermediates of GPI-APs are generated, which become subsequently re-acylated with stearic acid by the acyltransferase PGAP2, operating like Gup1 in yeast [60]. In both yeast and mammalian cells, GPI-APs are then transported from the *trans*-Golgi network (TGN) to PMs (for a review, see [61]). In addition, mammalian-polarized cells, such as epithelial or endothelial cells, sort and segregate their membrane proteins during their transport from the TGN to the cell surface along either the apical or basolateral cognate routes. One of the first signals for apical sorting postulated was the GPI anchor, since the majority of GPI-APs are, in fact, expressed at the extracellular leaflet of the apical PM domains [62,63,64,65,66]. In particular, in polarized epithelial cells, GPI-APs are predominantly sorted to the apical surface at the TGN under the control of specific factors by clustering into lipid rafts, which may operate as apical sorting platforms. Apical sorting of GPI-APs seems to depend on their accumulation at lipid rafts formed at the TGN membrane as defined by their resistance towards extraction by cold non-ionic detergent in the course of arrival and subsequent remodeling of the GPI anchor fatty acids for the expression of long-chain saturated fatty acids at the Golgi apparatus [67]. However, association of GPI-APs with lipid rafts is assumed to be required but not sufficient for apical sorting [68,69,70,71]. Importantly, the experimental evidence hints to differences in the organization of GPI-APs at PMs between polarized and non-polarized cells. Distinct mechanisms engaged by GPI-APs for their selective transport along the secretory pathway with emphasis on the control of their export from the ER and/or the TGN, and multiple molecules and genes responsible for the polarized trafficking and transport of GPI-APs to distinct PM domains in polarized epithelial cells, have been proposed (for a review, see [72]). Interestingly, some native GPI-APs and GPI-anchored fusion proteins have been found to be transported across cells (transcytosis), such as the folate receptor from the basolateral to apical surface domains of intestinal [73] as well as retinal pigment epithelial cells [74] and tissues, such as LY6A across the blood–brain barrier [75]. The transcellular transport of GPI-APs, which seems to involve transcytosis via caveolae, may be useful for the oral delivery of protein therapeutics, such as insulin, as has previously been speculated [76].

## 3. Function, Clustering and Endocytosis of GPI-APs

Interference with the biogenesis of GPI-APs in the course of the deletion of the corresponding genes has revealed that their cell surface expression is of critical importance for a variety of physiological pathways, among them for the vitality of yeast [77], for the survival and virulence of parasitic protozoa in the host [78], for the cell wall synthesis (see below, Section 4.1.1), morphogenesis, development of root meristems, germination, shoots and pollen tube growth in plants [79,80], and for the embryogenesis [81], neurogenesis and immune responses [82,83], fertilization [84], skin development [85], bone formation, brain functions and prion disease pathology [86] in mice. In particular, defective deacylation of the inositol ring in PGAP1-knock-out mice led to neonatal death associated with otocephaly and malformation of the jaw in most cases, whereas the few survivors displayed a considerably reduced body weight and male sterility [86]. Moreover, naturally occurring somatic mutations in hematopoietic stem cells of humans, among them PIG-A (catalytic subunit of glucoaminyltransferase) and autosomal recessive mutations in humans, among them PIG-H (glucosaminyltransferase), PIG-M (catalytic subunit of mannosyltransferase I), PIG-V (mannosyltransferase I), PIG-S (regulatory subunit of GPI-T), PIG-G (catalytic subunit of EtN-P transferase II) and PIG-N (EtN-P transferase I), i.e., in genes encoding enzymes of GPI biosynthesis, which consequently lead to GPI-deficient cells, have been correlated to several human diseases, such as the acquired hemolytic disease, paroxysmal nocturnal hemoglobinuria [87,88], defective immune response [89], dysregulated homeostasis of blood coagulation and neurological function [90], hyperphosphatasia mental retardation syndrome [91], multiple congenital anomalies-hypotonia-seizures syndrome [92], schizophrenia-associated cognitive dysfunction [93], neurological syndromes encompassing fetal akinesia, developmental delay and autism [94], early onset epileptic encephalopathy, intellectual disability and seizures [95,96], defects in the musculoskeletal system and nose, cleft palate and cognitive disability [97], schizophrenia in elderly subjects [93] and development of a rare blood group system with associated developmental deviations [98].

Replacement of the GPI anchor of cellular scrapie prion protein (PrP^C^) for a transmembrane domain caused its trafficking from lipid rafts to non-raft areas of the PMs and protection of this non-raft version of PrP^C^ against conversion into the misfolded scrapie form (PrP^SC^) following expression in cultured scrapie-infected neuronal cells [99]. This apparent protection has subsequently been shown to be due to targeting of the transmembrane cellular and GPI-anchored PrP^C^ to distinct membrane domains. Strikingly, infection of neuronal cells missing endogenous prion protein with wild-type GPI-anchored PrP^C^ or with purified amyloid fibrils formed by anchor-less PrP^C^ led to the generation of misfolded PrP^SC^ with the GPI-anchored but not anchor-less prion variants. Thus, apparently, the GPI anchor seems to determine the targeting of PrP^C^ to PM rafts with concomitant conversion into PrP^SC^ and formation of amyloid fibrils [100]. Consequently, GPI anchorage as signal for lipid raft localization of PrP^C^ has been hypothesized to be a prerequisite for its propagation as prion. However, the assumption that anchorage by GPI and residence at lipid rafts of PrP^C^ supports its misfolding into PrP^SC^ and the development of infectious scrapie disease is apparently in contradiction to the observation that PrP^C^ with missing GPI anchor was produced and propagated with significantly higher efficacy in vivo with accompanying elevated risk for the transmission of prion diseases [101]. Additional investigations must clarify the role of the GPI anchor for the replication of PrP^C^ and its targeting to the site of conversion to PrP^SC^. Together, these findings hint to a critical role of GPI anchorage and its specific structure in the (patho)physiological function of GPI-Aps during development and disease. The potential relevance of disturbances in the mechanisms regulating the biosynthesis, intracellular trafficking, and PM organization of GPI-APs for the development of diseases has been discussed elsewhere (for a review, see [66,101,102,103]).

Although the GPI anchor represents a complex and evolutionarily well conserved structure, a unifying concept for its function(s) has not developed so far. It may be argued that the energy required for the biogenesis of GPI-APs must be associated with an advantage for survival versus the energetically less demanding production of transmembrane proteins via natural selection during evolution. In fact, certain proteins have been reported to exert their function only if anchored with GPI (rather than a transmembrane domain). One prominent example is provided by Cripto-1 (CR-1), which acts as a co-receptor for the ligand Nodal, a member of the family of TGF-ß growth factors and regulates developmental downstream signal transduction by Nodal in vertebrates [104]. Importantly, the co-receptor function of CR-1 in supporting Nodal signaling in both cell-autonomous and non-cell-autonomous fashions has been demonstrated to depend on membrane-anchorage and intercellular transfer in order to exert its paracrine activity, which is mediated by a GPI anchor rather than a typical transmembrane domain or a soluble version, which has been constructed by recombinant truncation of the carboxy-terminus or mutation of the GPI signal sequence [105]. These findings argue for membrane anchorage of CR-1 by GPI being a prerequisite for its function as a Nodal co-receptor (for a review, see [106]). However, other proteins seem to fulfill their physiological role as both GPI-AP and transmembrane protein, among them CD4 [107] and EPCR [108]. In greater detail, high-level expression of a fusion protein between CD4, the major cellular receptor for the (envelop glycoprotein gp120 of) human immunodeficiency virus (HIV), and GPI attachment signal sequence of DAF in HeLa cells resulted in their sensitivity to gene transfer with a recombinant HIV vector comparable to native transmembrane CD4 [107]. The transmembrane receptor EPCR acts as a cofactor for protein C (PC) signaling, which functions as an anticoagulant and cytoprotective pathway, involved in multiple diseases. Upon binding of activated PC (APC) in endothelial cells, EPCR mediates stimulation of APC-mediated protease-activated receptor (PAR) downstream signaling, which is known to be frequently reduced in inflammatory diseases with accompanying downregulated cell surface expression of EPCR and cellular APC activity [108]. Interestingly, GPI-anchored EPCR with deleted transmembrane domain (EPCR-GPI) was found to be stably inserted into PMs of EPCR-deficient endothelial cells upon incubation in time- and concentration-dependent fashion and was found to restore PC signaling, as reflected in APC-triggered cleavage of PAR1/3 and PAR1-dependent AKT phosphorylation. These data are compatible with full functionality of EPCR-GPI in the engineered cells [108]. Moreover, CD4 as well as EPCR was targeted to lipid rafts, which express caveolin-1, arguing for maintenance of the functional state of GPI-APs upon their GPI anchor-mediated targeting to and insertion into cavelin-1-expressing caveolae at the target cells [107,108].

The data available for the GPI-APs, 5′-nucleotidase or CD73, Thy-1 (which possibly represents the smallest cell surface protein and nevertheless acts as both key ligand and receptor in the regulation of signaling and differentiation processes in cells of endodermal, mesodermal and ectodermal origin, apparently by dampening downstream signal transduction [109]) and human placental alkaline phosphatase (PLAP) argue for a very short distance or even direct contact of the protein moiety with the outer leaflet of the PM bilayer [110,111,112], with the glycan core moiety of the GPI anchor being positioned between the phospholipid head groups and the surface of the protein moiety in a compact and tightly folded configuration [113] or in a glycan-binding pocket at the protein moiety itself [114]. The very close positioning of the GPI-AP protein moiety above the lipid bilayer may enable transmission of compositional and structural alterations of the PM outer leaflet to the protein moiety with consequences for its shape and function.

GPI-APs have been found to be intrinsically associated with specific biochemically defined domains at the PMs, the so-called (detergent-insoluble and glycolipid-enriched) microdomains or lipid rafts [115]. A single long-chain-saturated fatty acid in the glycerophospholipid, (glyco)sphingolipid or ceramide moiety of GPI-APs, introduced in the course of fatty acid remodeling in concert with cholesterol at high concentration seems to be critical for the accumulation of GPI-APs in lipid rafts. Within rafts, the transient formation of GPI-APs in monomers, in homodimers provoked by GPI-GPI interactions, in nanodomains or -clusters in conjunction with (glyco)sphingolipids, cholesterol and elements of the actin cytoskeleton and in larger clusters or microdomains of liquid-ordered state surrounded by components of the cytoskeleton has been demonstrated [116,117,118,119].

**Figure 2:**
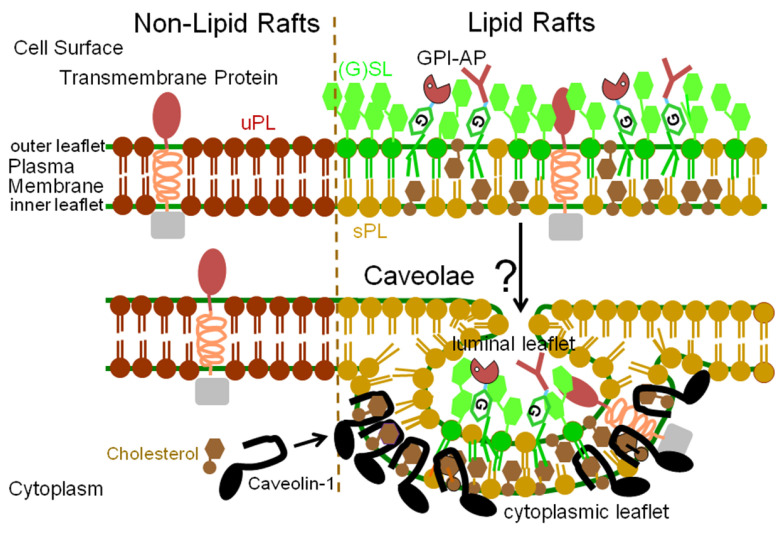
Localization of GPI-APs and their endocytosis (via caveolae) at lipid rafts, which are made up of (glyco)sphingolipids (GSL) at the outer leaflet of the PM bilayer, caveolin-1, phospholipids with saturated fatty acyl chains (sPL), and cholesterol at the inner leaflet. In contrast, non-lipid rafts predominantly contain transmembrane proteins and phospholipids with unsaturated fatty acyl chains (uPL). Lipid rafts are thought to act as nucleation sites for the biogenesis of caveolae upon invagination of the PMs into the cell interior. Following incision and fusion, the formed membrane vesicles would harbor the typical components of lipid rafts, among them cholesterol in concert with caveolin-1 (enriched at the cytoplasmic leaflet of the membrane bilayer), glycosphingolipids (enriched at the luminal leaflet) and GPI-APs. Their GPI anchor may become inserted in the luminal leaflet of the membrane bilayer and their protein moieties located in the vesicle lumen. Not only endocytosis, but also transcytosis of GPI-APs may involve caveolae. Experimental data supporting this view are currently intensively disputed (for a review, see [121,122]).

In contrast, GPI-APs residing outside lipid rafts are thought to freely diffuse within the plane of the PM outer leaflet as monomers. The interaction between membranes and GPI-APs has been studied in detail using intestinal and placental alkaline phosphatases upon reconstitution into supported model systems and atomic force microscopy (for a review, see [120]). Typically, in the course of phase separation, GPI-APs have been detected in the most (liquid) ordered rather than fluid membrane domains under physiological conditions but found to alter their distribution with temperature and to elicit changes in the size of those microdomains (Figure 2).

Lipid rafts operate as short-lived molecular assemblies of different lipidic and proteinaceous molecules, among them GPI-APs, within a rather restricted PM area and thereby trigger the transient local accumulation of higher molecular order as well as regional compartmentalization of those molecules with resulting formation of typical “membrane landscapes” [123,124]. For a given cell type, the dynamics of its actin network underlying the PMs may determine and control the temporal and spatial stabilization of nanoclusters of membrane constituents up to the total “membrane (protein) landscape”. Apparently, PM heterogeneity is caused by several distinct mechanisms rather than a single one. At least one of these seems to rely on assemblies of specific membrane (glyco)phospholipids, membrane proteins and cortical cytoskeletal elements and another apparently does not depend on the cytoskeleton [125]. Putative physiological or pathological roles of these (nano)clusters or -domains are critically dependent on their lifetime within the PMs of a given cell type, on the effect of their clustering (for a sufficient period of time) of relevant GPI-APs and on their structural and functional characteristics. This type of PM organization has been termed “active actin-membrane composite” cell surface to emphasize the critical impact of the cytoskeleton on the lateral mobility of PM proteins including the GPI-APs [124]. This model of the dynamic coupling of the inner leaflet to the underlying cortical actin network leaves open the nature of the apparently intimate interactions between (the peripheral proteins and phospholipids of) the inner leaflet and (the phospholipids and GPI-APs of) the outer leaflet of the PM bilayer.

Taken together, it is reasonable to assume that small transient lipid raft-free GPI-AP homodimers operate as basic building blocks for the assembly into and physiological functioning as more extended and more stable lipid raft-harboring GPI-AP homooligomers at the outer PM leaflet. Subsequently, they undergo clustering and immobilization into larger multi-component assemblies, which are enriched in and clustered together with cytoskeletal actin filaments as well as phosphatidylserine at the inner PM leaflet, and are possibly identical with typical lipid rafts. Thus, the combined theoretical simulations and experimental findings argue for the operation of a straightforward molecular mechanism of nanocluster- and domain formation, which is capable of inter-leaflet or transbilayer coupling in both directions, i.e., for signal transmission from the extracellular milieu via the PM inner leaflet to the cell interior and vice versa from the cytoplasm via the cell surface to the environment [125,126].

In fact, based on the known interaction of GPI-APs and short-lived actomyosin complexes occurring within lipid rafts and the demonstration of binding of complexes of GPI-AP-induced lipid rafts, actin filaments and anionic lipids of those rafts to (and stimulating) a number of actin-nucleating and regulating factors, among them formins and N-WASP, in addition to acylated src-family kinases alone or via their effectors cortactin and abl-kinases, it has been hypothesized that the cortical cytoskeleton represents the main target of full-length GPI-APs for the formation of specific “membrane landscapes” and downstream signaling initiated thereby to morphogenetic and guidance mechanisms of cells expressing the relevant GPI-APs (e.g., uPAR, T-cadherin) at PMs [127,128]. An interesting model has been proposed for the induction of massive alterations in the shape and three-dimensional configuration of “PM landscapes” in response to pronounced rearrangements of the cortical cytoskeleton in microdomains of extended and stabilized complexes of oligomers, multimers or nanoclusters of GPI-APs [129]. This multimerization of GPI-APs may be provoked by the binding of multivalent ligands which leads to coalescence of rafts and to increases in their lifetime. Consequently, the type of ligand and GPI-AP involved in the biogenesis of lipid rafts may ultimately determine the shape of a given cell, in general, and the specific “landscape” of its PMs, in particular, by induction of invaginations (as nucleation site for the GPI-AP enriched endosomal compartment “GEEC”) or protrusions (ruffles, spikes) at PMs [129]. In addition, the “active actin-membrane composite model” suggests organization of transmembrane proteins and GPI-APs at the outer phospholipid leaflet of PMs with the aid of a template formed by cortical acto-myosin activity which interacts with GPI-APs across the phospholipid bilayer under involvement of cholesterol and phosphatidylserine at the inner leaflet [130]. Then, local liquid-ordered nanodomains may be generated which in turn, in concert at a mesoscale, may contribute to the “overall” shape and specific “landscape” of PMs.

Visualization of the topography of PMs with accompanying spatiotemporal distribution of clusters of transmembrane proteins and receptors (e.g., IgM antigen receptor), which may also encompass full-length GPI-APs, in conjunction with the underlying actin cytoskeleton, has recently been accomplished for B cells of the Ramos Burkitt’s Lymphoma line using “lattice light-sheet microscopy” in combination with tailored “custom-built 4D image analysis” [131]. Fascinatingly, PMs were found to build up dynamic networks of protruding ridges which span individual microvilli and are equipped with a portion of the clustered B cell antigen receptors. Importantly, the dynamics of the ridge topology and the mobility of the clustered membrane proteins were correlated [131], perpetuating the above view that ligand binding to transmembrane- and GPI-anchored receptors may induce the biogenesis of protrusions and invaginations of PMs, i.e., of its specific “landscape”, with accompanying signaling to the cell interior.

However, GPI-APs and GPI lipids per se, i.e., in the absence of interactions with the cortical actin cytoskeleton underlying the PMs, may also manage to form specific “landscapes” of protrusions and invaginations. PMs of eukaryotic unicellular parasites, such as *Trypanosoma cruzi* and *Leishmania major*, exhibit a variety of GPI-APs, among them mucin and lipophosphoglycans, respectively, which have been conserved during eukaryotic evolution to guarantee ongoing interaction with their hosts (for a review, see [132]). Thus, GPI-APs and GPI lipids have been proposed as universal building units for evolutionary creation of the huge structural variety of the “PM landscapes” of trypanosomatids, and this could also apply to other protozoans and mammalian cells.

**Figure 3:**
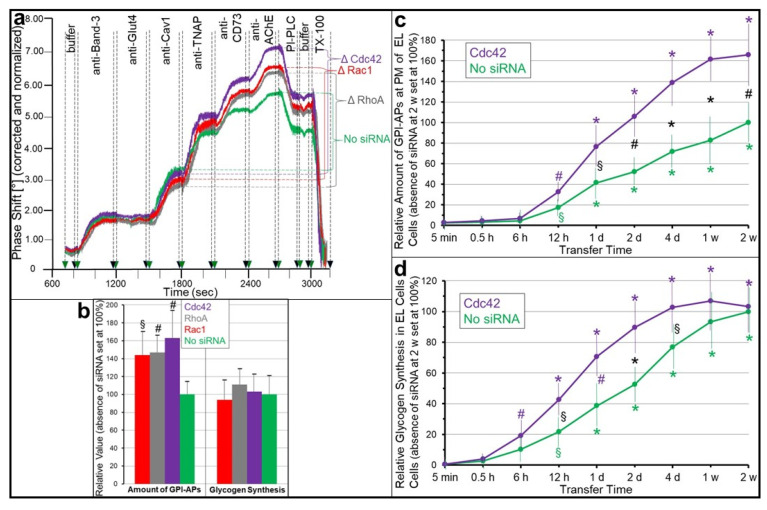
Inhibition of internalization of GPI-APs during transfer from human adipocytes and stimulation of glycogen synthesis in the ELCs. Transwell co-cultures were run (37 °C, absence of serum and BSA, (**a**,**b**), 2 weeks; (**c**,**d**), transfer times as indicated) with human donor adipocytes (of lipid-loading stage IV) and GPI-deficient acceptor ELCs in the insert and bottom wells, respectively, in the absence (**a**–**d**, No siRNA) or presence of siRNAs (30 nM) directed against Cdc42 (**a**–**d**), Rac1 (**a**,**b**) or RhoA (**a**,**b**). Subsequently, PMs were prepared from the ELCs of the bottom wells, then coupled to chips by ionic/covalent capture and finally analyzed for the expression of membrane proteins by SAW biosensing. (**a**) Phase shifts Δ in response to injection of anti-TNAP, anti-CD73 and anti-AChE antibodies (1800 to 2700 sec) were measured as summation signals and are indicated by horizontal hatched lines and brackets for each incubation. (**b**–**d**) The experiment was repeated three to four times (different transwell co-cultures). Relative amounts of total transferred GPI-APs at PMs of ELCs (summation signals) (**b**,**c**) as well as relative glycogen synthesis (0.1 mM glucose) (**b**,**d**) in the absence (set at 100%) or presence of the siRNAs are given. Significant differences between the presence and absence of siRNAs at identical transfer times (**b**–**d**, black symbols) as well as between the various transfer times and the 5-min times for presence and absence of Cdc42 siRNA each (**c**,**d**, turquoise and green symbols, respectively) are indicated (means ± S.D.; * *p* ≤ 0.01, ^#^ *p* ≤ 0.02, ^§^ *p* ≤ 0.05).

Internalization of endogenous as well as transferred GPI-APs is mediated by the GPI-AP-enriched endosomal compartment (GEEC) rather than clathrin-coated vesicles or caveolae, which are engaged in the endocytosis of transmembrane proteins (for a review, see [133,134,135,136,137]). In the experiment presented here, the endocytosis of full-length GPI-APs (i.e., GPI-APs with the complete GPI anchor remaining attached rather than proteolytically or lipolytically cleaved versions; see below, Section 4.1.2 and Section 4.1.3) by acceptor cells, here GPI-deficient erythroleukemia cells (ELCs), was investigated. These were released from the donor cells, here human adipocytes, upon incorporation into micelle-like complexes, consisting of (lyso)phospholipids and cholesterol (see below, Section Release into Micelle-Like Complexes), rather than into extracellular vesicles (EVs) or by proteolytic or lipolytic release, and then transferred to the ELCs. Endocytosis was monitored using transwell co-culture and chip-based biosensing for the amount of the transferred full-length GPI-APs left at the PMs (phase shift of horizontal surface acoustic waves (SAW) induced by binding of GPI-APs antibodies to the chip surface with the immobilized recovered PMs) upon incubation in the absence or presence of silencing of critical components of the GEEC, i.e., RhoA, Rac1 and Cdc42 [138,139,140,141,142]. The experiment (Figure 3) was performed as has been described previously [143], with the only difference being the time point of initiation of silencing at the start rather than following transfer of the GPI-APs.

siRNAs directed against RhoA, Rac1 and Cdc42 caused significant increases in antibody-induced SAW phase shifts compared to the absence of siRNAs (Figure 3a, green curve; Figure 3b, left panel, green bar) in that ranking order of increasing efficacy for the GPI-APs TNAP, CD73 and AChE, but not for the transmembrane proteins Band-3, Glut4 and Cav1. This demonstrated time-dependent accumulation of the transferred GPI-APs, but not of typical and atypical transmembrane proteins, at PMs of the acceptor ELCs during blockade of their endocytosis (and under concomitant ongoing transfer of GPI-APs from the donor adipocytes) by the GEEC-specific siRNAs, which reached a maximum (1.6-fold vs. no siRNA) at 2 weeks (Figure 3c, shown only for Cdc42).

Previously, it has been shown that the residence at PMs of ELCs of the GPI-APs which have been transferred from human adipocytes was correlated to stimulation of glycogen synthesis in the acceptor cells [143]. Inhibition of endocytosis of the GPI-APs by different approaches following their transfer, among them silencing of a key component of their endocytosis by the GEEC, led to prolonged expression of the GPI-APs at PMs, as confirmed here (Figure 3a,c), and induction of the anabolic state in the ELCs. Vice versa, restoration of endocytosis caused a decrease in PM expression of the GPI-APs and downregulation of glycogen synthesis, with comparable kinetics [143].

Interestingly, maximal expression of GPI-APs at the PMs of the ELCs after two weeks of silencing of endocytosis (and ongoing transfer from the donor cells) (Figure 3b, left panel) was not correlated to any stimulation of glycogen synthesis in the acceptor cells (Figure 3b, right panel). Analysis of the corresponding time courses revealed that the impairment of endocytosis of GPI-APs upon silencing of Cdc42 (Figure 3c) led to significant elevations of glycogen synthesis only between 12 h and 4 d of blocked endocytosis and ongoing transfer of GPI-APs, but not earlier or later (Figure 3d).

These data confirmed previous findings [143] that (i) residence at PMs of transferred GPI-APs depends on the rates of both their transfer from donor to acceptor cells and internalization via the GEEC, that (ii) impaired internalization of the transferred GPI-APs by inactivation of the GEEC leads to prolongation of their residence at PMs, and that (iii) residence at PMs of the transferred GPI-APs is required for transfer-induced glycogen synthesis. The apparent discrepancy in the time courses between maximal residence at PMs (at two weeks) and maximal glycogen synthesis (four days to two weeks) with inactivated GEEC (Figure 3c,d, turquoise lines) in contrast to parallel increases of PM residence and glycogen synthesis with functional GEEC (Figure 3c,d, green lines) may be explained as follows: (i) localization of GPI-APs at PMs is required but not sufficient for induction of glycogen synthesis and (ii) only a limited amount of GPI-APs at PMs is coupled to the machinery of induction of glycogen synthesis, leading to its maximal activation prior to saturation of the PM residence of the GPI-APs and thus to partial uncoupling of PM expression and induction of glycogen synthesis by full-length GPI-APs.

Furthermore, the similar kinetics of the persistence of both PM expression of transferred GPI-APs and upregulation of glycogen synthesis upon silencing of critical components for GEEC function during (Figure 3) and after transfer [143] demonstrate that the stimulation of basal glycogen synthesis by (endogenous as well as transferred) GPI-APs depends on their localization at rather than endocytosis from the PMs of the (acceptor) cells. These findings emphasize an important functional aspect of (a subset of) GPI-APs: The induction of intracellular effects, here metabolic signaling to the glycogen synthesis machinery, critically depends on their residence at the PMs and is thus regulated by their endocytosis (in concert with their de novo biosynthesis and transfer from donor cells). It will be an important but challenging task to identify the nature of those GPI-APs which mediate anabolic signaling (i.e., stimulation of glycogen and lipid synthesis).

## 4. Release of GPI-APs

The possibility that the (major) physiological role of GPI anchorage of cell surface proteins is based on their constitutive and/or regulated release into the extracellular space has already been suggested in the course of elucidation of the structure of the GPI anchor [144]. Initially, cleavage at the carboxy-terminus of the protein moiety or within the GPI anchor has been suggested as the only molecular mechanism for the release of the GPI-AP protein portion in yeast, protozoa, and mammalian cells. Later, the release of intact GPI-APs harboring the full-length GPI anchor with involvement of either vesicular or non-vesicular structures has also been demonstrated for various mammalian cell types. It is well conceivable that the function of the released GPI-APs is critically determined as to whether they operate as hydrophilic anchor-less or amphipathic anchor-containing entities as well as by the structure of the retained entities. The putative fates of GPI-APs following their biogenesis and targeting to PMs in mammalian cells were proposed with astonishing precision almost three decades ago in a seminal review article (see ref. [145]).

### 4.1. Cleavage of the GPI Anchor

The principle of membrane attachment of GPI-APs intuitively hints to the GPI anchor itself providing preformed cleavage sites for (endogenous or exogenous) enzymes that catalyze the release of the protein moieties into the extracellular space [146,147]. Interestingly, a number of GPI-anchored enzymes, receptors, binding and transport proteins, signaling proteins and extracellular matrix proteins have been shown during decades of investigation to display considerable alterations in their catalytic activity, binding characteristics or signaling capacity between the soluble anchor-less and the amphipathic full-length GPI-anchored version [148,149,150,151,152,153,154,155,156]. This raised the possibility that the full-length GPI-APs act as either functionally inactive or active versions immediately following their biogenesis and thereafter undergo activation and inactivation, respectively, in the course of cleavage of their anchor during subsequent cell growth and development. However, it has remained difficult to discriminate at the cellular level or in vivo as to whether the functional characteristics of full-length GPI-APs compared to their anchor-less versions are due to the modification of the GPI per se or anchorage at PMs per se or both in concert.

#### 4.1.1. Transglucosylation of the Glycan Core

In the yeast *Saccharomyces cerevisiae*, a considerable portion of its about 60 GPI-APs is transferred and coupled to the cell wall and consequently is engaged in cell wall integrity and assembly (for a review, see [157,158,159]), among them Cwp2p [159] and Tip1p [160], whereas others remain anchored at the PMs [157] or reside at both locations, among them Gas1p [161] and Gce1p [162]. During transfer of GPI-APs to the cell wall, the glucosamine of the glycan core together with PI is eliminated and the protein moiety becomes coupled to the ß-1,6-glucan of the cell wall via the three mannose residues remaining left at the glycan core [163] in the course of a transglucosylation reaction involving Cwg6/GPI-3 [164]. Interestingly, the decision between targeting the cell wall or PMs critically depends on the sequence upstream of the GPI attachment (ω) site [165]. Different (positive or negative) signals for cell wall targeting [166,167] or targeting by default [168] have been proposed so far. Moreover, more recent studies have shown that the type of inositolphosphorylceramide in the GPI lipid portion is involved in the retention of GPI-APs at PMs [166,169]. Finally, Cwh43p and the genetically related Ted1p, which encode proteins engaged in the elimination of EtN-P from the second mannose residue of the glycan core [166] as well as Dcw1 and Dfg5p, putative mannosidases [167], may operate as components of a sorting machinery, localized at or near the PMs, which functions by recognizing the amino acid sequences upstream of the ω-site, and transfers a specific class of GPI-APs from the PMs to the cell wall.

#### 4.1.2. Proteolytic Cleavage

**Figure 4:**
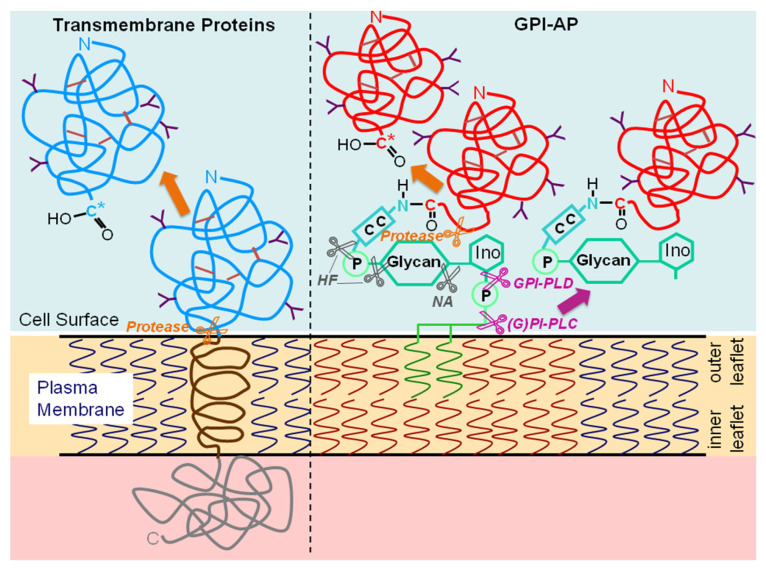
Different modes of enzymic release of membrane proteins from PMs by cleavage of their transmembrane domain or GPI anchor. Proteases cleave either at the juxtamembrane region of transmembrane proteins or at the extreme carboxy-terminus of GPI-APs and thereby cause the release of the extracellular domains of both the former and the latter (with no residue of the GPI glycan core left) into the extracellular compartment. The site of the proteinaceous cleavage determines whether the released protein moiety is truncated at its carboxy-terminus by a single (C) or a few amino acids (C*) and may therefore affect the functional state of the released GPI-AP. GPI-specific phospholipases (PL) of specificity C or D (GPI-PLC/D) cleave at distinct positions within the phosphodiester bridge of PI, which thereby results in different cleavage products, i.e., the release of the complete protein moiety together with the phosphoinositolglycan (PIG) as PIG-protein (by GPI-PLC) or with the inositolglycan moiety as inositolglycan-protein (by GPI-PLD), leaving behind at the PMs the corresponding diacyl or acylalkylglycerol and phosphatidate moieties, respectively. Certain chemicals, such as hydrogen fluoride (HF) and nitrous acid (NA), which have been used during the initial characterization of GPI anchorage by non-enzymic means, cleave within the glycan core of the GPI anchor of GPI-APs and cause the separation of the protein moiety from the terminal EtN-P residue in the course of dephosphorylation (HF) or from the non-acetylated GlcN in the course of deamination (NA). Importantly, those chemical methods with their unique cleavage specificities and the resulting release of the GPI-AP protein moieties can only be used in vitro rather than in living organisms. Ino, inositol; CC-N-H, ethanolamine.

Proteolytic cleavage causes release of the extracellular domains of a large number of transmembrane proteins, but so far has been reported only for a few GPI-APs (for a review, see [170]), among them for the GPI-anchored prion protein [171], urokinase type of the plasminogen activator receptor (uPAR) [172], the tumor-associated MHC class I polypeptide-related sequence A (MICA) [173] as well as the NKG2D ligand ULBP3 [174] by metalloproteases of the ADAM family and the human kidney Tamm–Horsfall glycoprotein at a site within the extracellular domain near the cell surface [175]. The specificity of proteolytic cleavage has the clear-cut advantage of the option of individual control of the release of GPI-APs by a unique protease. However, it does not explain the development of GPI-APs during evolution vs. transmembrane proteins, which are accessible for proteolytic attack, too (Figure 4). Thus, additional biochemical characteristics enabling physiological roles which cannot be fulfilled by typical transmembrane proteins have to be assumed as the driving force for the introduction of GPI-APs during the evolutionary step from pro- to eukaryotes.

#### 4.1.3. Lipolytic Cleavage

In contrast to above, the number of findings about lipolytically cleaved GPI-APs has increased steadily over the past three decades, among them trypanosomal VSG during differentiation of the bloodstream to the procyclic forms of *Trypanosoma brucei* [176]; human alkaline phosphatase (AP) and ecto-5′-nucleotidase or CD73 upon incubation of human lymphocytes with tumor necrosis factor-α [177], which has been correlated with elevated adenosine levels and extracellular purine metabolism in the blood of adults compared to newborns, fostering an anti-inflammatory immunological status [178,179]; uPAR from breast cancer cells in the extracellular matrix, which has been correlated with neoplastic transformation, tumor growth and mortality [180]; carcinogenic embryonic antigen (CEA) [181]; renal dipeptidase from kidney proximal tubules [182,183]; tissue non-specific alkaline phosphatase (TNAP) [184]; growth arrest specific 1 (GAS1) [185]; and CD14 [186].

Various enzymes which cleave the GPI anchor at different sides of the phosphodiester bond of PI, i.e., of type C and D, and thereby liberate the diacylglycerol or phosphatidic acid portion of the GPI anchor, respectively, from the protein moiety together with a terminal (phospho)inositolglycan residue, i.e., harboring or lacking a (cyclic) phosphate residue, respectively (see Figure 4), have been identified.

Bacterial PI-PLCs secreted from *Bacillus thuringiensis*, *Bacillus cereus*, *Staphylococcus aureus*, *Clostridium novii* and *Listeria monocytogenes* are capable of hydrolyzing mammalian GPI anchors in addition to their “natural” substrate PI (for a review, see [187]).

Parasitic protozoal GPI-PLCs in the cytoplasm of *Trypanosoma brucei* and *Leishmania major* manage to convert membrane-associated amphiphilic GPI-APs into their soluble hydrophilic versions. Cleavage by both bacterial and trypanosomal (G)PI-PLCs has commonly been regarded as the typical biochemical criterion for the detection of GPI anchorage of GPI-APs since their initial description. However, it must be taken into consideration that (i) bacterial PI-PLCs fail to cleave GPI anchors which are acylated (e.g., palmitoylated) at the position 2 of the *myo*-inositol ring [188,189,190], (ii) glycosylations in the neighborhood of the carboxy-terminus of the protein moiety of GPI-APs may impair cleavage by bacterial PI-PLC [188], (iii) the type of phospholipids and in consequence their fluidity and packing-together along the membrane may affect the cleavage efficacy of bacterial PI-PLCs [125,190,191] and (iv) the lipolytically cleaved hydrophilic version of GPI-APs may not be immediately released from the cell surface due to peripheral interactions [192,193,194,195].

Various PI-PLC isoforms of the unicellular ciliates *Paramecium caudatum* and *Paramecium tetraurelia* have been found to be associated with the corresponding cell surface and released into the culture medium together with lipolytically cleaved GPI-APs, among them PLC2 and PLC6, and thus may be responsible for the release of GPI-APs into the circulation of infected organisms [196,197].

Glycerophosphodiesterases GDE3 and GDE2, which are six-transmembrane ecto-phosphodiesterases of mammalian cells displaying a large catalytic ectodomain, have been shown to lipolytically cleave (with type C and D specificity, respectively) the GPI anchor of uPAR in breast cancer cells [180] and of RECK and glypican-6 during neuroblastoma development [198], respectively, and are said to have a role in cancer metastasis and neuronal differentiation, respectively. Interestingly, GDE2 does not digest uPAR, which argues for the specificity of the GDEs [199].

Insulin-/sulfonylurea (SU)-induced GPI-PLC was demonstrated to release a number of GPI-APs, among them AP, lipoprotein lipase, the glycolipid-anchored cAMP-binding ectoprotein and cAMP phosphodiesterase Gce1, CD73 and a membrane dipeptidase, from the intact surface of mammalian insulin target cells upon their challenge with insulin, growth factors and anti-diabetic SUs [186,200,201,202,203,204,205,206,207]. With the help of a specific inhibitor of the insulin-/SU-induced GPI-PLC [205], a major role of this enzyme in metabolic insulin signaling and action in primary rat adipocytes has been excluded, whereas it has been found to be indispensable for the insulin-mimetic and insulin receptor-independent effects of the anti-diabetic blood glucose-lowering sulfonylurea drug (SU) glimepiride [203,204,205].

The *Drosophila* hydrolase Notum has been identified as a GPI-PLC that cleaves the glypican DLP as well as glypican-3 and the morphogenic Wnt and Hedgehog proteins [208], causing their release from the intact cell surface [209].

Mammalian GPI-PLD (GPLD1) is an abundant serum protein [210,211,212,213], synthesized by the liver, which can associate with high density lipoproteins (HDL) in an inactive state [214] or with cellular membranes (of mammalian liver) [215] and cleaves the GPI anchor of a number of GPI-APs in vitro, but only in the presence of detergents, e.g., upon solubilization of PMs [216]. Thus, importantly, serum GPLD1 does not release native GPI-APs from the intact cell surface into the circulation. However, strikingly, it is active at the ER membranes, possibly due to weak or incomplete insertion of the GPI anchors during their biosynthesis [217]. So far it remains open as to whether serum GPLD1 manages to cleave specific GPI-APs at intact cell surfaces in mammalian tissues, but only under tightly controlled conditions, which prevents the unwanted general release of all GPI-APs into the circulation. Alternatively, serum GPLD1 (in complex with HDL) may remove those full-length GPI-APs which had been released from tissue cells into circulation by some (spontaneous or unspecific) mechanisms to bypass possible deleterious effects based on their amphipathic nature, such as aggregation, solubilization of PMs and sticking to or unspecific insertion into the PMs of blood cells, vascular endothelial cells and tissue cells (see below, Section 4.2). A cell-associated GPI-PLD has been described to specifically release neural cell adhesion molecule (NCAM) from differentiating myoblasts [218], uPAR from ovarian cancer cells [219] and CEA from human colon cancer cells [220]. Interestingly, the serum concentration of GPLD1 has been reported to be elevated in old mice in response to exercise and to be correlated to the strength of their cognitive function [221]. Moreover, upregulated levels of GPLD1 in blood have also been measured for healthy elderly humans. Systemic administration of GPLD1 to aged mice caused compensation of the age-induced regenerative and cognitive losses in the course of triggering signaling cascades downstream to cleaved serum GPI-APs. It has been concluded that those blood factors released from their GPI anchorage by the hepatokine GPLD1 transduce signals from the liver to the brain and thereby mediate beneficial effects for neurogenesis and cognition in the elderly [221].

PGAP6 (TMEM8A) represents a cell-surface-associated phospholipase A_2_ which is expressed in a wide variety of mammalian cells and displays a narrow substrate specificity towards GPI-APs (CRIPTO, glypican-3, prostasin, SPACA4, contactin-1) [222]. The release of CRIPTO, which acts as a co-receptor for the morphogenic factor Nodal, plays a critical role in an early stage of embryogenesis. For this, in a first step, PGAP6 eliminates one fatty acyl chain from the GPI anchor, leading to CRIPTO equipped with and anchored at PMs by a single fatty acid moiety only. In a second step this residual fatty acid becomes liberated in spontaneous fashion, or the lysophosphatidic portion of the GPI anchor is removed with the aid of a PLD, such as GPLD1 [222].

For many mammalian GPI-APs which have been found to be lipolytically released from cells and tissues in vitro or in vivo in the basal state or under certain (patho)physiological conditions or in response to certain hormones and stimuli, among them epidermal growth factor [223], interleukin-2 [224], adrenocorticotropic hormone [225], thyroid stimulating hormone [226] and nerve growth factor [227], the relevant GPI-specific phospholipases (GPI-PLs) have not been identified so far as holds true for the molecular mechanisms of their activation. To overcome this limitation, a systematic whole-organism screen for GPI-cleaving enzymes has been performed using transgenic mice which express GPI-anchored GFP overall in the body [228]. It led to the detection of GPI-AP-releasing activities in the interstitial fluids of several organs, such as testis and pancreas, but left open their cleavage specificities except for a mannosidase activity which was attributed to the testicular isoform of the angiotensin converting enzyme (tACE) [229]. Importantly, release of GPI-APs from intact membranes in vitro by tACE did not rely on its intrinsic protease activity and was blocked by the presence of cholesterol in the PMs. Unfortunately, reproduction of these results has failed so far [230,231].

#### 4.1.4. Physiological Roles of Proteolytic and Lipolytic Release of GPI-APs

The expression of released GPI-APs and GPI-cleaving enzymes in eukaryotic organisms raises the question about the physiological relevance of the release of GPI-APs, in general, and the function of the released protein moieties, in particular. So far, experimental evidence for the following options has been presented:

(i) Removal of GPI-APs from the cell surface for degradation and functional inactivation or for activation has been demonstrated for GPI-anchored cell adhesion molecules under the control of PLC2 and PLC6 in *Paramecium tetraurelia* [196], for brain TNAP in the extracellular neuronal matrix [184], for GAS1 released from neuronal progenitors and glomerular mesangial cells into the cerebrospinal fluid (CSF) and plasma of rats [185] and for CD14 lipolytically released from cultured RAW264 and microglial cells under accompanying downregulation of the signaling induced by peptides, which accumulate in prion, Alzheimer’s and Parkinson’s diseases [186]. Thus, agents that affect the lipolytic release of certain GPI-anchored (anti)inflammatory signaling molecules in the brain may be envisaged as novel remedies for neurodegenerative diseases.

(ii) Change of the conformational and functional characteristics of the GPI-AP protein moiety due to its liberation from the GPI anchor and/or (the intimate neighborhood to) the PMs as has been demonstrated for Gce1p of the yeast *Saccharomyces cerevisiae* and its kinetics and specificity of cAMP-binding [154].

(iii) Development of novel properties of PMs upon removal of GPI-APs has been demonstrated for the tACE-cleaved GPI-AP coat of sperms which facilitates its binding to the zona pellucida of the egg [229,232].

(iv) Generation of cleavage products from the GPI anchor has been demonstrated for glypican-3 upon release by mammalian notum, which operates as antagonist of Wnt receptor and thereby as a negative regulator of Wnt signaling [209] and for PIG-proteins cleaved off from GPI-APs of insulin target cells by insulin-/SU-induced GPI-PLC [146,233].

(v) Generation of soluble GPI-AP protein moieties with specific physiological function has been demonstrated for CD73, Gce1, Thy-1, AP and CD16 receptors and the insulin-/SU-induced GPI-PLC in insulin target cells upon challenge with insulin [186,199,200,201,202,203,204,205,206,207,208,209,234,235,236,237,238], which following their appearance in human circulation and certain tissue cells and their subsequent interaction with cognate receptors or uptake should result in corresponding downstream signaling. The same holds true for human GPIHBP1 and CD59 with its complement regulatory function in nerves, kidney and vasculature which become impaired in the course of diabetes [239,240,241,242,243], presumably as a result of its non-enzymic glycation in dependence on the glucose concentration as well as exposure time [242,243] and lipolytic release from cell surfaces into interstitial fluids, serum [244,245] and urine [246]. Interestingly, the insulin-/SU-induced GPI-PLC has been shown to be upregulated by glucose in rat adipocytes [204], explaining the positive correlation between hyperglycemia or diabetes and glycated CD59 in blood and arguing for it as a potential biomarker for the prediction, monitoring, prognosis and/or stratification of vascular diabetic late complications.

Glypican-4 (Gpc4), a coreceptor of growth factors, such as Wnt, fibroblast growth factors and Hedgehog in mammals [247,248,249], represents an additional example for the release and function of GPI-AP protein moieties with the soluble anchor-less Gpc4 capable of interacting with the unoccupied insulin receptor and stimulating insulin signaling [250]. Importantly, the detection of soluble Gpc4 in serum in correlation with BMI and insulin sensitivity may be attributed to its lipolytic release from adipose tissue by GPLD1, which is known to cleave its GPI anchor [193,209]. Taken together, lipolytically cleaved soluble Gpc4, the release of which into human serum is fostered by elevated insulin levels in response to insulin resistance and obesity, seems to operate as an insulin-sensitizing adipokine and to counteract impaired insulin signaling via a feedback loop. Additional aspects about GPI-specific PLs and the physiological relevance of the lipolytic release of GPI-APs have been discussed recently (for a review, see [251]).

Interestingly, a recent study demonstrated that the GPI attachment signal sequence (see above, Section 2) can determine the mechanism of release of the corresponding GPI-AP, i.e., proteolytic vs. lipolytic [252]. For this, the GPI attachment signal sequence of the cellular PrP^C^, a GPI-AP which plays a critical role in transmissible neurodegenerative and fatal prion diseases, has been exchanged for that of Thy-1 in cells and transgenic mice. This hybrid PrP^C^ has turned out to be less susceptible to proteolytic release compared to authentic PrP^C^. Importantly, transgenic mice expressing the hybrid PrP^C^ and infected with prions displayed a significantly delayed terminal disease, altered MAPK signaling and reduced microglia/astrocyte activation compared to wildtype mice [252]. Thus, the nature of release of GPI-APs may affect the (patho)physiological pathway initiated by the released protein portions.

This view was reinforced by previous findings that a GPI-AP may be released by both proteolytic and lipolytic release in simultaneous fashion [253]. GPI-anchored heparan sulfated glypican-4 (GPC-4) was released from the surface of cultured mouse primary astrocytes by both proteolytic and lipolytic cleavage, presumably exerted by ADAM9 metalloprotease and a GPI-PLC/D, respectively, to a major and minor portion, respectively. Since both releasing mechanisms result in the production of the active version of GPC-4, which promotes synapse maturation and function, it remains to be clarified whether and how the differential release of GPC-4 contributes to a regulatory pathway of neuron–glia communication involved in synaptogenesis.

### 4.2. Release of Full-Length GPI-APs

**Figure 5:**
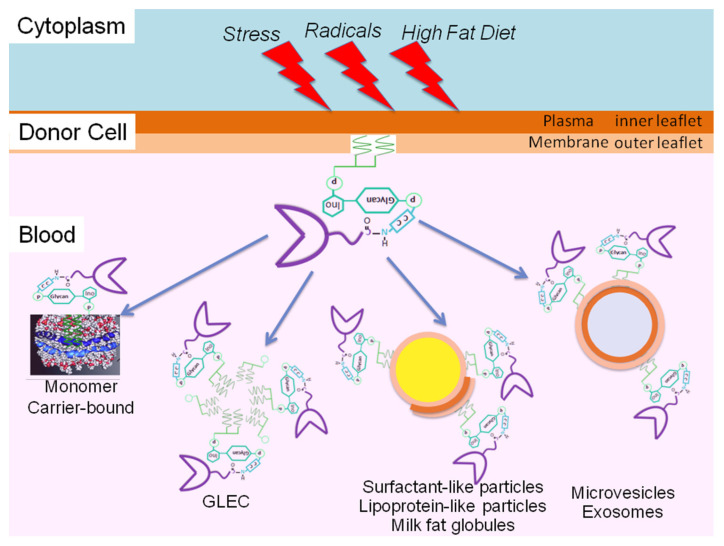
The different structural entities and assemblies involved in the release of full-length GPI-APs with the complete GPI anchor remaining attached from the PMs of donor cells are represented by specific monomeric GPI-AP carrier- or binding-proteins (GPI-binding proteins) or micelle-like GPI-AP complexes constituted of (lyso)phospholipids and cholesterol (GLEC) or lipoprotein-like particles (LLPs) surrounded by phospholipid bi- and/or monolayers or extracellular vesicles (EVs), such as exosomes and microvesicles, formed by phospholipid bilayers. They are released from the outer leaflet of the PM bilayer of donor tissue or blood cells of mammalian organisms into extracellular compartments, such as blood or interstitial spaces, spontaneously or in response to specific endogenous or exogenous stimuli.

Based on theoretical considerations, i.e., the need for protection of the hydrophobic GPI anchor fatty acyl chains against access of the hydrophilic aqueous milieu of extracellular compartments, several options for the appropriate configuration of structures and assemblies harboring full-length GPI-APs are conceivable: phospholipid bilayers (vesicular, planar), phospholipid monolayers (lipoprotein-like particles, multilamellar particles), lipid-containing micelle-like complexes, lipid-free homo- and heteromultimeric protein/GPI-AP aggregates and GPI-AP carrier-/binding-proteins. In fact, each of these strategies of masking of the GPI anchor seems to be realized in biological systems (Figure 5).

#### 4.2.1. Release into Exosomes

The AMP-hydrolyzing enzyme ecto-5′-nucleotidase or CD73 was the first GPI-AP, for which the release from the surface of eukaryotic cells into small membrane vesicles, at those times termed microvesicles, has been described more than four decades ago [254]. Subsequently, small phospholipid/cholesterol-containing membrane vesicles of 50 to 200 nm diameter have been visualized and demonstrated to mediate the removal of the transferrin receptor from the surface of sheep reticulocytes following the receptor-mediated endocytosis of transferrin and recycling of the transferrin receptor back to the PMs prior to their maturation into erythrocytes in vitro [255,256,257]. These observations led to the hypothesis that those vesicles, now termed exosomes [258,259], operate as waste containers to get rid of superfluous (e.g., in case of reticulocyte maturation into erythrocytes), inactivated, defective or “harmful” proteins from the interior as well as the surface of eukaryotic cells (for a review, see [260]). The release by exosomes was subsequently confirmed for a large number of full-length GPI-APs, among them AChE, decay accelerating factor (DAF, CD55) and membrane inhibitor of reactive lysis (MIRL) [261], lymphocyte function-associated antigen 3 (LFA-3) [262], PrP^C^ [263,264], Gce1, CD59 and AP [262,265] for a variety of mammalian blood and tissue cell types, such as platelets [266,267], T cells [268,269], enterocytes [270,271], B lymphocytes [272], dendritic cells [273] and a number of tumor cell types (see e.g., [274]).

The diversity of GPI-APs released into exosomes and the multitude of cell types capable of releasing exosomes have led to the “educated guess” that the release of GPI-APs via exosomes does not only play a role in the removal of inactive or unwanted GPI-APs, which is supported by the following findings: Monocyte-derived dendritic cells release exosomes with abundant GPI-anchored MHC molecules CD55 and CD59, suggesting a possible function in the presentation of (glyco)lipidic antigens and in complement regulation [275], in particular, and hinting to a role of exosomal GPI-APs in immune function, in general. PrP^C^, GPI-anchored at the surface of peripheral blood cells and operating as a precursor for the pathogenic conformational variant scrapie prion protein (PrP^Sc^), was released into exosomes from cultured THP-1 monocytes [276] as well as from cultured cortical neurons together with the GPI-AP ceruloplasmin [277], suggesting a (patho)physiological function of exosomal GPI-AP release at neuronal synapses. NKG2D-ligands, expressed as both transmembrane proteins and GPI-APs, were released from the PMs of malignant placental, ovarian and prostate cancer cells by incorporation into exosomes [278,279] with resulting impairment of effective NKG2D-dependent immune responses. This is thought to foster the distribution of tumor cells and creation of a tumor microenvironment, as has been deduced from clinical studies reporting elevated levels of specific GPI-anchored and presumably exosome-associated NKG2D-ligands in sera and tumors of cancer patients with poor prognosis (for a review, see [280,281]). The GPI-AP GPI-80, initially identified on human neutrophils, was found to be released into exosomes from several tumor cell lines, such as PC3 prostate cancer cells, and detected in the plasma of prostate cancer patients [282]. Furthermore, GPI-80 was also recovered with the synovial fluid of rheumatoid arthritis patients, presumably upon its release from neutrophils into exosomes, which is under positive and negative control of TNF-α and antioxidants, respectively [283].

Vesicles morphologically similar to exosomes have been identified in seminal plasma, consequently termed prostasomes or epididymosomes, and found to be enriched with sphingomyelin and cholesterol as well as the full-length GPI-APs CD59, CD55 and CD52 at their surface [284] (for a review, see [285]), each of them seemingly supporting the sperm function. This also holds true for the GPI-APs high-affinity folate-binding protein and P34H [286,287], which could have a bacteriostatic function in the epididymal duct by depriving folate-requiring bacteria of folate (for a review, see [288]).

Exosomes are formed in the cytoplasm within multivesicular bodies (MVBs) in the course of inward budding of their membranes into their luminal space, subsequent incision and fusion of the MVB membranes with the resulting entrapment of cytoplasmic components as well as the incorporation of membrane proteins of the MVBs, among them GPI-APs, into the interior and surface, respectively, of so-called intralumenal vesicles (for a review, see [289]). Targeting of the MVBs to and fusion with the PMs causes exocytosis of their luminal content and thereby release of their contents, the intraluminal vesicles, now termed exosomes, together with the full-length GPI-APs, which remain embedded at their outer membrane leaflet, into the extracellular space [290]. GPI-APs destined for intraluminal vesicles may arrive at the MVB membranes either en route from the ER via the Golgi apparatus along the secretory pathway or from the PMs via the endocytic/degradative pathway. These dual modes of biogenesis explain how GPI-APs which had or had not been expressed at the cell surface were both efficiently released from eukaryotic cells in exosomes [289,290]. Since, however, most GPI-APs become exposed at the PMs during their lifetime, the endocytic pathway may represent the predominant mechanism for their exosomal release. Taking this route, endocytic vesicles with the protein moieties of GPI-APs facing the luminal membrane leaflet fuse with the MVB membranes under maintenance of the specific orientation of the GPI-APs during the subsequent formation of the intraluminal vesicles, which critically depends on Cos proteins (in yeast) and tetraspanins (in mammalian cells) and the Endosomal Sorting Complex Required for Transport (ESCRT) machinery and ubiquitin ligases (i.e., Rsp5-Sna3 complexes in yeast and MARCH ligases in mammalian cells) [291]. With regard to the last step, the release of exosomes from the luminal contents of MVBs following their exocytosis seems to be under the control of the GPI-AP tetherin, which has been demonstrated to keep the newly generated exosomes attached at the PMs in a clustered state [292]. On the basis of the differential release of exosomes in correlation to the (patho)physiological state of the donor cells and despite their loading with soluble and transmembrane proteins, mRNAs, miRNAs and lipids, valid arguments have been put forward in favor of the potential use of GPI-APs as biomarkers, in particular for cancer [293] and metabolic diseases [294].

#### 4.2.2. Release into Microvesicles

In addition to the release by exosomes, GPI-APs, among them CD73, have been recovered from so-called microvesicles (size 100 nm to 1 μm) with “right-side out” sealed configuration and the protein moiety of GPI-APs facing the outer leaflet of the PMs in active state in response to certain extrinsic cues [295] or spontaneously [296]. Microvesicles are generated by a multitude of human cell types, among them platelets, erythrocytes, endothelial cells and tumor cells [295,296,297,298,299,300,301,302], through sequential budding, incision, and fusion and final shedding of certain areas of the eukaryotic PMs, possibly at lipid rafts with clustered GPI-APs [292], into body fluids of healthy probands and patients [299,302], among them blood [297,298,299,300,303], saliva [304], synovial fluid [305], seminal fluid [306] and urine [307]. Release of microvesicles can be stimulated by numerous (patho)physiological factors (e.g., cytokines, hormones, endotoxins) and situations (e.g., oxidative stress, mechanical force, hypoxia, elevated blood flow, hyperglyceridemia, hypercholesterolemia, hyperglycemia, hyperinsulinemia, inflammation, pregnancy, pre-eclampsia, coagulation) [295,296,297,298,299,300,301,302,303,304,305,306,307,308,309,310,311]. However, release may also happen spontaneously in response to ageing as shown for erythrocytes, which release microvesicles enriched in GPI-APs, including CD55 and CD59, during prolonged storage [302,312] or to alterations in the phospholipid or cholesterol composition of the PMs of blood cells [300,302,313,314]. Importantly, the release of microvesicles rather than that of exosomes is driven by (patho)physiological increases in cytosolic Ca^2+^ (for a review, see [315,316]) and oxidative stress [317], e.g., in the course of development of (cardio)vascular and cancer disorders. This is accompanied by exposure of phosphatidylserine at the outer leaflet of the PMs together with the GPI-APs and accompanying abrogation of the flippase- and scramblase-determined phospholipid asymmetry between the outer and inner PM leaflets at sites where microvesicular budding takes place (for a review, see [318]). Elevated cytosolic Ca^2+^ seems to be sensed by TMEM16F, a Ca^2+^-activated chloride channel, which induces the reorganization of the cytoskeleton [317] with resulting blebbing of the PMs. Shedding of microvesicles seems to represent the predominant mode of the vesicle-mediated GPI-AP release from the PMs (for a review, see [318,319,320]).

It is of substantial physiological relevance that the release of full-length GPI-APs by exosomes and microvesicles, often commonly referred to as extracellular vesicles (EVs) to avoid the difficult differentiation on the basis of the currently used experimental protocols [321], share the regulation by various environmental conditions, among them mechanical stress (e.g., shearing forces, blood flow), oxygen deprivation, metabolic state (e.g., high glucose and/or insulin) and viral infection (e.g., HIV-1) as well as by factors, among them oxygen radicals, receptor agonists, certain drugs (e.g., glimepiride) and hormones (e.g., insulin). Thus, release of GPI-APs into both exosomes and microvesicles does not operate constitutively in most cell types studied, among them primary rat and cultured mouse adipocytes [322,323,324,325,326]. Interestingly, adipocyte EVs are made up of specific subsets of luminal proteins, transmembrane proteins and GPI-APs, among them Gce1 and CD73, which cooperate in the degradation of cyclic adenosine monophosphate (cAMP) via AMP to adenosine [327,328], the mRNAs coding for glycerol-3-phosphate acyltransferase-3 (GPAT3) and fat-specific protein-27 (FSP27), each of them contributing to stimulation of lipid synthesis and lipid droplet biogenesis, as well as the microRNAs, miR-16 and miR-22, which have been implicated in the coordination of lipid metabolic pathways [329,330]. Challenge of the adipocytes with certain physiological stimuli, such as hormones, palmitate or reactive oxygen species as well as the anti-diabetic sulfonylurea drug glimepiride has been reported to trigger the release of EVs harboring CD73 and Gce1 [322,327], which was significantly more pronounced with large cells compared to small ones [331,332,333]. Strikingly, upon exposure to EVs which had been released from large adipocytes, small rat adipocytes displayed considerably elevated lipid synthesis, exceeding that of large adipocytes incubated with EVs from small ones [332,333,334,335,336]. On that experimental basis it has been suggested that adipocyte-derived EVs manage to coordinate lipid metabolism between large “lipid-full” and small “empty” adipocytes within the same adipose tissue depot or fat pad by transferring (some of) the constituent components, among them GPI-APs, and thereby shifting the burden of lipid loading from the former to the latter.

#### 4.2.3. Release into Non-Vesicular Structures and Assemblies

The following experimental findings obtained during the last two decades have focused on the apparently labile cell surface anchorage of GPI-APs with resulting amphitropic localization at both PMs and extracellular compartments, as a consequence of their seemingly spontaneous non-enzymic release from the outer leaflet of the PMs due to their pronounced overall amphiphilic character (i.e., large hydrophilic protein moiety coupled to amphiphilic GPI):

(i) The forces required for extraction from supported phospholipid bilayers are lower for the GPI-AP AP compared to typical transmembrane proteins as measured with atomic force microscopy [337,338].

(ii) The interaction forces between planar distribution of mixtures of phospholipids and cholesterol and the long-saturated fatty acyl chains typically present in GPI anchors are considerably lower compared to typical transmembrane domains as revealed by Langmuir film technique and fluorescence microscopy [339,340].

(iii) The interaction of GPI-APs with artificial biological membranes, which strongly depends on the length and saturation of the GPI fatty acyl chains and membrane phospholipids, is less tight compared to transmembrane proteins, as demonstrated by Fourier transform-infrared reflection-absorption spectroscopy [341,342].

(iv) Certain GPI-APs are released from eukaryotic cells in response to age, size and the differentiation or metabolic state, with the complete GPI remaining attached [343,344,345].

(v) Those full-length GPI-APs are detected in extracellular aqueous compartments, such as body fluids or culture media, embedded into membranous structures, such as vesicles and lipoprotein-like particles (LLPs), or assembled into lipid-containing micelle-like complexes and lipid-free multimers or associated with GPI-binding proteins (for a review, see [346]).

(vi) Full-length GPI-APs incorporated into detergent- or (lyso)phospholipid-/cholesterol-containing mixed micelles can be inserted into liposomes as well as PMs of intact cells in vitro under conservation of their activity, a process previously called cell surface painting [347,348].

(vii) GPI-APs (in contrast to transmembrane proteins) are capable of being transferred between phospholipid bilayers, as has initially been shown for the GPI-AP AchE upon incubation of protein-free sealed dimyristoylphosphatidylcholine liposomes with human erythrocytes [349], and this has subsequently been extended to other GPI-APs and to the transfer from intact donor to acceptor cells (for a review, see Müller and Müller, submitted manuscript).

##### Release into LLPs

The first studies dealing with the non-vesicular release of full-length GPI-APs relied on intestinal AP, even prior to its characterization as a GPI-AP, and its detection in human lymph and serum [350] as well as in rat intestinal lumen and mucosal surface upon high fat feeding [351] or cholecystokinin administration [351,352]. Those findings have suggested the embedding of the diacylglycerol moiety of the GPI anchor of intestinal AP in phospholipid layers which surround non-vesicular lipid-filled particulate entities. Subsequently, intestinal AP with the complete GPI anchor remaining attached has been found to be associated with the apical microvillar membrane surface of the intestinal lumen in surface-active and phospholipid-containing so-called surfactant-like particles (SLPs), harboring the surfactant-specific protein SP-B and mediating the surfactant-like appearance and surface tension-lowering property, but lacking a typical phospholipid bilayer structure [351,353]. SLPs are thought to be generated by sequential budding, vesiculation, fusion and final shedding of specific protrusions from the PMs, which are equipped with a subset of the GPI-APs expressed by the donor cells [354]. Secretion of SLPs together with full-length GPI-APs from the brush border membranes of enterocytes has been reported to be upregulated by the absorption of dietary lipids and free fatty acids [355], subsequent to endocytosis of the GPI-APs into lipid raft-like structures [356,357]. Thereafter, the possible range of the physiological roles of the GPI-APs released into small SLPs has been expanded from gas exchange in pulmonary epithelia to transepithelial transport of dietary fatty acids and triacylglycerol across the enterocyte, which is mediated by the release of intestinal AP and other GPI-anchored hydrolases in extrapulmonary epithelia [358]. Furthermore, intestinal SLPs have been detected in rat and human serum [359], which was unexpected considering their release from the enterocyte apical surface into the intestinal lumen. Consequently, it has been suggested that the serum concentration of SLPs reflect mucosal integrity, in general, and the intactness of the tight junctions between the enterocytes from which they have been derived, in particular [360,361].

Milk fat globules (MFGs) consist of lipid droplets (LDs) of 1–10 μm diameter, which resemble the structure of typical cytoplasmic LDs of mammalian adipocytes (i.e., a core of cholesterylester and triglycerides covered by a monolayer of phospholipids and unesterified cholesterol, for a review, see [362,363,364]) and are surrounded by a phospholipid bilayer membrane with intercalated members of the family of apolipoproteins [365,366]. MFGs are thought to be assembled and secreted into the milk by mammary secretory cells lining the mammary gland (e.g., breast) duct epithelia [367]. The phospholipid bilayer membranes are apparently derived from the PMs of the secreting donor cells, presumably in the course of consecutive blebbing, budding, incision, fusion and shedding processes. Thus the biogenesis of MFGs resembles the release of microvesicles from the PMs, thereby leading to enclosure of the MFG-specific LDs as their cytoplasmic content. In consequence, MFGs display at their surface, i.e., outer leaflet of the phospholipid bilayer, full-length GPI-APs which are typical for the donor cell [368], among them CD59 (protectin), CD55 (DAF) and HRF (C8bp homologous restriction factor) [369,370,371,372].

Interestingly, the outer leaflet of the MFG phospholipid bilayer apparently suffers from intrinsic instability since loss of GPI-APs from MFGs into the alveolar lumen soon after their secretion from mammary glands has been reported [373,374]. This was accompanied by the appearance of full-length GPI-APs, such as CD59, in aggregates of a heterogenous nature, which lack other (not GPI-anchored) MFG constituents. It has been speculated that these aggregates represent CD59-expressing membrane vesicles released from the MFGs into the environment, such as the alveolar lumen, in the course of spontaneous blebbing, budding, incision and fusion of their phospholipid bilayer. Alternatively, a non-vesicular mechanism is conceivable as well.

The demonstration that CD59 secreted into milk as a full-length GPI-AP is functionally active and of higher potency compared to its soluble (proteolytically or lipolytically cleaved) anchor-less counterpart [359,373,374] argues for the physiological relevance of the release of GPI-APs with complete anchor into MFGs. Nevertheless, at present it cannot be excluded that the expression of GPI-APs in MFGs of mammary milk simply represents a byproduct of their biogenesis at the sites of lipid rafts, which by nature express GPI-APs, and does not contribute to the specific functions exerted by the MFGs.

Lipid-modified, in particular, acylated proteins, among them the morphogens Wnt and Hedgehog, can be released from the producing cells and function at distant target cells after their transport along extracellular compartments in extracellular phospholipid-containing particles, so-called nodal vesicular particles (NVPs) [375,376]. They apparently resemble both SLPs and MFGs with regard to structure and biogenesis [377,378]. In detail, the membranes surrounding the NVPs are presumably derived from the PMs of the donor nodal cells in the course of blebbing, budding, incision, fusion and shedding from their apical microvilli. Thus, the biogenesis of NVPs is thought to rely on molecular mechanisms similar to those operating for the assembly and release of SLPs and MFGs as well as of membrane-enveloped mammalian viruses.

Although to our knowledge the presence of GPI-APs in NVPs has not been reported so far, the apparent similarities in structure and function of NVPs, SLPs and MFGs as carriers for lipid-modified proteins strongly suggest a role of NVPs in mediating the release and transport of cell type-specific subsets of GPI-APs. Their incorporation into the NVPs could occur late during biogenesis at the PMs of the donor cells, as is the case for SLPs and MFGs, rather than early at the level of the ER membranes, with the GPI-APs assembled as “secretory” proteins at the luminal face of the ER.

A variety of GPI-APs have been found associated with so-called lipoprotein-like particles (LLPs), among them lipophorin together with lipid-modified Hedgehog [375,376], which are presumably inserted into a phospholipid monolayer surrounding a core of neutral triacylglycerols and cholesterylesters together with members of the apolipoprotein family (for a review, see [379]). Thus, LLPs seem to represent an excellent environment for shielding of the amphipathic GPI anchors from access of the aqueous milieu and thereby enabling the surface expression of GPI-APs as well as of the fatty acyl and cholesteryl residues of lipid-modified proteins, such as Hedgehog and Wnt. It may be of relevance that cytoplasmic LDs, which from a structural point of view can be regarded as the intracellular counterpart of extracellular LLPs, have been reported to express a subset of GPI-APs in primary rat adipocytes [380]. Those apparently become translocated from the cell surface to the LDs in response to lipolytic stimuli, and have been proposed to contribute to the cAMP-mediated regulation of the lipolytic degradation of LDs [380,381].

LLPs may be generated during the biogenesis of typical lipoproteins in the course of separation of the luminal leaflet of the ER membranes with the inserted GPI-APs, cholesterol and apolipoproteins from the cytoplasmic leaflet. Subsequent to the filling-up of the interleaflet space with cholesterylester and triacylglycerol, the emerging LLPs finally bud into the ER lumen. Thereafter, the LLPs are directed into the canonical secretory pathway via inclusion into specialized transport vesicles which bud from the ER membranes. Following targeting to and fusion with the PMs of the Golgi-derived transport vesicles, LLPs become released from the cells into the extracellular space, such as the bloodstream [382], where the LLP-associated GPI-APs fulfill their physiological role. In addition to LLPs, HDLs have been demonstrated to act as carrier of CD59 [378], in course of its insertion into the phospholipid monolayer at the surface.

##### Release as Protein Oligomers, Multimers and Aggregates

There are additional mechanisms supporting the release of full-length GPI-APs from the surface of eukaryotic cells. They involve oligomeric/multimeric structures and aggregates in order to circumvent the need of vesicles or particles for burying or protection of the GPI lipid portion [383,384]. For this, the formation of homomeric or heteromeric oligomers or multimers of full-length GPI-APs of a unique or several type(s), respectively, has been proposed, with the fatty acyl chains of their GPI anchors packed together into the hydrophobic core of the aggregates and the hydrophilic headgroups of the GPI anchors and GPI-AP protein moieties remaining exposed at the surface of the aggregates. Overall, these aggregates display a configuration similar to that of mixed detergent-(lyso)phospholipid micelles, with the amphipathic GPI anchor constituents taking over the structural and functional roles of detergent molecules [385,386]. These multimeric aggregates may be formed by self-assembly, driven by the absence of exogenous (lyso)phospholipids. In fact, the GPI-APs CD59, CD55 and CD52 have been recovered with particulate materials from interstitial fluids, blood, seminal plasma and other body fluids [387,388]. Interesting data suggest that multimeric morphogen aggregates cannot fully substitute for LLPs in triggering the release, enabling the transport and mediating the function of Wingless and Hedgehog and presumably of additional GPI-APs during development, in case of heteromeric aggregates [376]. It remains to be clarified whether proteins distinct from the lipid-modified morphogens and GPI-APs are actually present in the high-molecular-weight multimeric morphogen aggregates, i.e., whether these are actually of heteromeric rather than homomeric nature [385].

##### Release for GPI-Binding Proteins

As another strategy to avoid access of the aqueous milieu to the saturated long-chain fatty acids of released GPI-APs, these may be buried within a hydrophobic cleft or groove of specific GPI-binding or GPI-carrier proteins. This type of interaction of GPI-APs in the monomeric state with specific type(s) or variant(s) of one or several fatty acid- or phospholipid-binding proteins has been suggested only very recently and so far delineated for, but not restricted to serum albumin [143,389] (for a review, see Müller and Müller, submitted).

##### Release into Micelle-Like Complexes

Three decades ago, it was shown that seminal plasma contains the full-length GPI-APs CD59, CD55 and CD52 [284,390,391,392] which were associated neither with prostasomes [284] nor assembled into lipid-free multimeric aggregates, but apparently formed as micelle-like complexes together with lipids [393]. Two sources are conceivable for GPI-APs released into those non-vesicular micelle-like complexes, which later turned out to contain (lyso)phospholipids and cholesterol [394], EV membranes and PMs. Irrespective of their origin, the release of the complexes could rely on the following multi-step mechanisms: (i) separation of the outer and inner phospholipid leaflets of the membranes, specifically at sites of high density of GPI-APs, (lyso)phospholipids and cholesterol, i.e., lipid rafts, (ii) bulbing of the outer leaflet together with the incorporated GPI-APs, (iii) incision and (iv) fusion of the monolayer consisting of GPI-APs, (lyso)phospholipids and cholesterol into complexes with micelle-like configuration. Thus, the apolar core constituted of the fatty acyl chains is presumably covered by the polar surface area formed by the lipid head groups and the GPI-AP protein moieties. It is tempting to speculate that the biogenesis of those complexes runs more efficiently with PMs than with EV membranes, given the more pronounced susceptibility of the former towards (oxidative and mechanical) stress and other environmental cues.

To study the possibility that full-length GPI-APs are released from eukaryotic PMs under certain (patho)physiological conditions, a chip- and microfluidic channel-based biosensor has been developed, which monitors any interaction at the chip surface by changes in phase and amplitude of horizontal SAW propagating over the chip surface [394,395]. Full-length GPI-APs were found to be released from rat adipocyte PMs immobilized onto the chip, which was dependent on the flow rate and composition of the buffer stream. Furthermore, similar complexes were identified in the incubation medium of primary rat adipocytes, in correlation to the cell size, and in rat as well as human serum [396]. Interestingly, the measured changes in the SAW phase shift, which reflect the amount of full-length GPI-APs in complex with lipids, as well as in the SAW amplitude, which reflect the viscoelasticity of the complexes, enabled the differentiation between lean and obese rats, normal and hyperinsulinemic rats as well as normal and hyperinsulinemic hyperglycemic rats [344]. It was concluded that chip-based biosensing for micelle-like complexes of full-length GPI-APs is indicative for the inherently labile anchorage of GPI-APs at PMs and hence their susceptibility for release in response to (intrinsic/extrinsic) metabolic cues and may, therefore, be useful for monitoring and stratification of (pre-)diabetic states.

**Figure 6:**
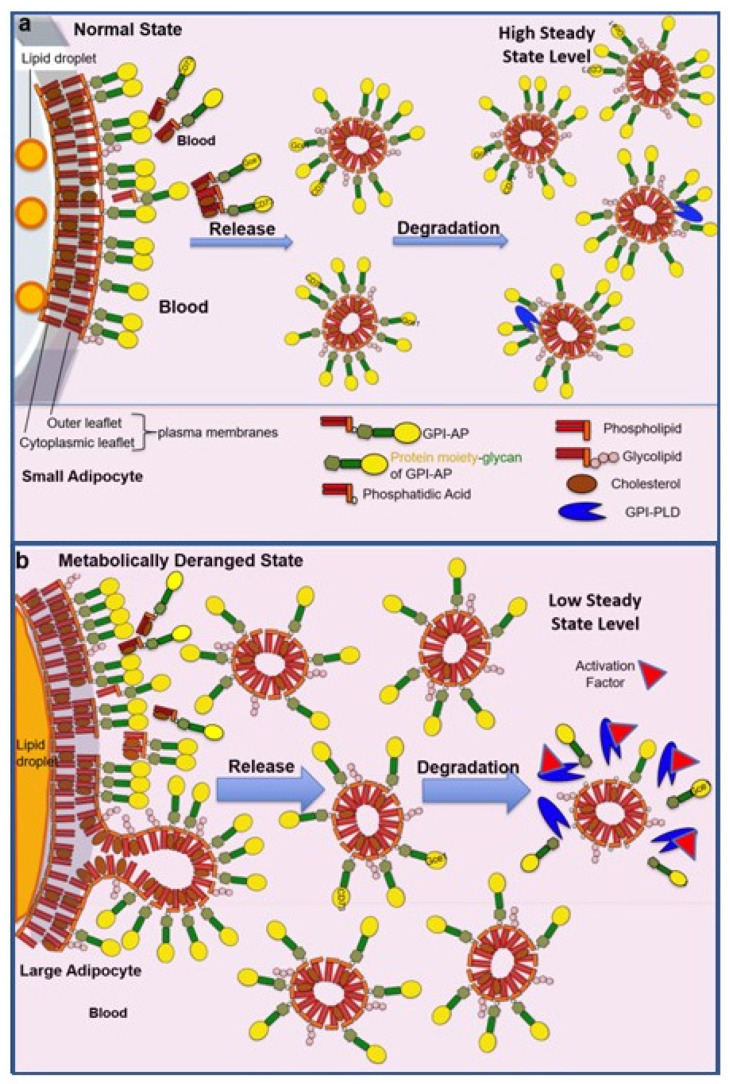
Hypothetical model for the release of full-length GPI-APs from adipocytes and their degradation by serum GPI-PLD (GPLD1), which both determine the steady-state concentration of full-length GPI-APs at micelle-like serum complexes in the normal (Panel a) and metabolically dysregulated state (Panel b). The upregulation of the release of full-length GPI-APs into micelle-like complexes because of destabilization of the PMs of the relevant blood or tissue cells is overcompensated by the increase of the GPLD1 activity. GPLD1 activity is controlled by its interaction with a so-called activating factor (red triangles) and ultimately leads to lower steady-state serum concentrations of the complexes than those in the normal state. The rapid removal of the full-length GPI-APs from the blood stream by the elevated GPLD1 activity is presumably of tremendous importance on the basis of their pronounced amphiphilic character. In fact, micelle-like GPI-AP complexes have been demonstrated to cause lysis of adipocytes upon incubation, as measured as the release of lactate dehydrogenase into the medium [394]. Differential interaction of the activating factor with the GPLD1, which is dependent on the nature of the donor cells and tissues as well as the extent of destabilization of their PMs, may explain the elevated serum GPLD1 activity in the metabolically deranged state.

This possibility has been tested by a comparison of the signatures of horizontal SAW that were generated by full-length GPI-APs in the course of their specific capture by and subsequent dissociation from the chip-based biosensor between those from rat serum and those reconstituted into lipidic micellar structures in vitro [394]. The signatures obtained strongly argued for the expression of full-length GPI-APs in serum in micelle-like complexes in concert with (lyso)phospholipids and cholesterol. Both the reconstituted and the rat serum complexes were highly sensitive toward mechanical forces. Furthermore, full-length GPI-APs reconstituted into micelle-like complexes represented efficient substrates for cleavage by the serum GPLD1 [395,396,397]. These findings raised the possibility that the upregulated release of full-length GPI-APs into micelle-like serum complexes from metabolically deranged cells is compensated by elevated GPLD1 activity. In fact, serum GPI-PLD activity towards full-length GPI-APs in micelle-like complexes, but not in detergent micelles, turned out to be positively correlated to early states of insulin resistance and obesity in genetic and diet-induced rat models as well as to body weight in humans [394]. These data suggest that the serum GPLD1 activity measured with GPI-APs in micelle-like complexes is indicative of enhanced release of full-length GPI-APs from relevant tissue cells into the circulation as a consequence of early metabolic derangement as well as ageing in rats and humans [345,394]. In extension of these findings, (i) the release of the full-length GPI-APs CD73, AP and CD55 from isolated adipocyte PMs, as monitored in a “lab-on-the-chip” configuration, (ii) their release from isolated rat adipocytes into the incubation medium and (iii) the lipolytic cleavage of their GPI anchors in serum were found to increase with age (3–16 weeks) and body weight (87–477 g) of (healthy) donor rats [394,396]. In contrast, the amount of full-length GPI-APs in rat serum, as determined by chip-based biosensing, turned out to decline with age/body weight [397]. These correlations suggested that certain age-/weight-induced alterations (of certain biophysical/biochemical characteristics) of the PMs are responsible for the release of full-length GPI-APs, which is counteracted by elevated GPLD1 activity in serum. A model for the release and putative deleterious effects of micelle-like GPI-AP complexes in the circulation of mammalian organisms and their prevention by degradation in dependence on the metabolic state is presented (Figure 6).

## 5. Conclusions

Since the introduction of the ingenious fluid-mosaic model for biological membranes by S.J. Singer and G.L. Nicolson in 1972 [398,399], it has generally been assumed that their constitutive components, i.e., (glyco)phospholipids, cholesterol, and integral membrane proteins, are characterized by both (differential) lateral mobility along the plane of the membrane and simultaneously stable anchorage at the bilayer structure. The latter ensues resistance of both the lipids and the (typical, e.g., class I) transmembrane proteins against release into cytoplasmic or extracellular compartments due to spontaneous or (patho)physiological (induced) processes. So far, this paradigm has been taken to guarantee the identity and integrity of cells (and organelles), in general and of PMs, in particular. More specifically, removal of typical, i.e., non GPI-anchored, membrane proteins from the PMs has been thought to rely solely on their (i) endocytic uptake with subsequent intracellular recycling or degradation (for a review, see [133,134,135,136,137,400,401]), (ii) enzymic cleavage at the extracellular proteinaceous domains with resulting liberation from the cell surface in order to fulfill signaling functions [170,171,172,173,402,403,404,405] and (iii) release into eVs in order to get rid of unwanted or non-functional membrane proteins by shedding of PMs as microvesicles (for a review, see [315,316,317,318,319,320]) or endocytosis/exocoytosis of PMs as exosomes (for a review, see [260]) or to mediate the vesicle-dependent transfer of typical integral membrane proteins from donor to acceptor cells with (patho)physiological consequences [406,407] (for a review, see [408,409]). Each of these canonical pathways for the removal of typical transmembrane proteins from PMs of eukaryotic cells has also been demonstrated to operate for GPI-APs, however with involvement of GEEC instead of clathrin-coated vesicles and of GPI-PLs instead of proteases. Thus, it seems likely that the apparently straight-forward mode of the release from PMs of GPI-APs through a small set of phospholipases of different cleavage specificity, but of unique substrate specificity, in contrast to the involvement of a large panel of proteases with unique cleavage specificity, but different substrate specificity for the release of transmembrane proteins, together with the possibility of their intercellular transfer via a variety of distinct non-vesicular structures, containing or lacking lipids, rather than only through EVs [410,411,412], represents the main driving force for the development and conservation during evolution of GPI-APs vs. canonical transmembrane proteins. The (patho)physiology of the intercellular transfer of GPI-APs will be addressed in a complementary forthcoming review (Müller and Müller, submitted).

## Data Availability

Not applicable.
